# Eating Problems in Autistic Females and Males: A Co-twin Control Study

**DOI:** 10.1007/s10803-021-05198-z

**Published:** 2021-07-22

**Authors:** Karl Lundin Remnélius, Janina Neufeld, Johan Isaksson, Sven Bölte

**Affiliations:** 1 Center of Neurodevelopmental Disorders (KIND), Centre for Psychiatry Research, Department of Women’s and Children’s Health, Karolinska Institutet & Stockholm Health Care Services, Region Stockholm, Gävlegatan 22B, Floor 8, 113 30 Stockholm, Sweden; 2 Department of Neuroscience, Child and Adolescent Psychiatry Unit, Uppsala University, Uppsala, Sweden; 3 Child and Adolescent Psychiatry, Stockholm Health Care Services, Region Stockholm, Stockholm, Sweden; 4 Curtin Autism Research Group, Curtin School of Allied Health, Curtin University, Perth, Western Australia Australia

**Keywords:** Autism, Gender differences, Eating, Co-twin control design

## Abstract

This study investigated the association between autism and self-reported eating problems and the influence of gender on the association, in a sample of adolescent and adult twins (N = 192). Autistic traits and autism diagnosis were associated with both total and specific eating problems, including selective eating and sensory sensitivity during mealtimes. Interaction effects indicated a stronger association between autistic traits and total eating problems in females, as well as more difficulties with eating in social contexts among autistic females. In within-pair analyses, where unmeasured confounders including genes and shared environment are implicitly controlled for, the association was lost within monozygotic pairs, which might further indicate a genetic influence on the relationship between autism and eating problems.

**Supplementary Information:**

The online version contains supplementary material available at 10.1007/s10803-021-05198-z.

## Introduction

Autism spectrum disorder, henceforth *autism*, is defined by qualitative challenges in social interaction and communication along with restricted, repetitive patterns in behavior, interests and activities (RRBIs) (American Psychiatric Association, 2013), and is generally understood as the extreme end of a quantitative distribution of autistic traits in the general population (Constantino, 2011). The condition is considered highly heritable, with heritability estimates ranging from 64 to 91% (Tick et al., 2016). Twin data suggest that autism and autistic traits share origins, in form of identical genetic factors (Lundström et al., 2012). Autism has been linked to an increased occurrence of eating problems, including selective eating, characterized by eating a restricted range of foods, sometimes depending on food characteristics such as color or texture, and food neophobia (i.e. avoiding or refusing new foods) (Råstam, 2008). Other reported problems involve behavioral inflexibility around mealtimes, e.g. requiring specific utensils to eat or performing rituals in mealtimes (for a review, see Sharp et al., 2013). Eating problems are more common among autistic than neurotypical children (Sharp et al., 2013), and have been reported among as many as 46% to 89% of autistic children (Ledford & Gast, 2006). Extending clinical findings in autism, recent studies indicate a link between dimensional autistic traits in the general population and eating problems (van ’t Hof et al., 2020; Wallace et al., 2018). Issues around eating have been suggested to be secondary consequences of the core symptom domains of autism (Johnson et al., 2014). Correspondingly, studies have reported associations with RRBIs, including insistence on sameness and sensory sensitivity (Johnson et al., 2014; Postorino et al., 2015; Zickgraf et al., 2020). While links between the social domain and eating behaviors are less consistently found (Johnson et al., 2014), a recent study yielded that autistic traits in this domain as well as in the RRBI domain were associated with increased food neophobia in children (Wallace et al., 2018). However, while associations are found, no causal relation has been established, i.e. that eating problems would arise as a consequence of autism.

A study investigating parental feeding concerns found no difference between parents of 24 neurotypical children and parents of 24 autistic children regarding the child’s first year of life (Provost et al., 2010). However, while feeding concerns decreased in the neurotypical group as the child grew older, concerns among parents to autistic children rather increased during the following years. Correspondingly, although research on eating problems in autism has primarily focused on children, studies indicate that these issues persist into adolescence and adulthood (Karlsson et al., 2013; Kuschner et al., 2015; Spek et al., 2020). For instance, autistic women and men self-report increased eating problems compared to neurotypical women and men, including selective eating and problems with simultaneous capacity when eating (Spek et al., 2020).

Eating problems may pose a variety of challenges for autistic people and their families. Qualitative findings suggest that problems related to food and mealtimes might limit participation in positive social interaction for autistic children and their families, where mealtimes are instead regarded stressful events (Marquenie et al., 2011). When compared to autistic children without such problems, autistic children who display co-occurring eating problems show elevated autistic traits, internalizing and externalizing problems, and their parents report increased parental stress (Postorino et al., 2015). In addition, repetitiveness and selective eating of mainly high-calorie foods might contribute to an elevated risk of obesity found in autism (Flygare Wallén et al., 2018; Kinnaird et al., 2019; Zheng et al., 2017). It should however be noted that qualitative findings propose that characteristics associated with autism such as rigidity and sensory sensitivity can be linked to restricted eating and clinical eating disorder (ED) diagnoses such as anorexia nervosa among autistic adults (Brede et al., 2020; Kinnaird et al., 2019).

Researchers have suggested a link between autism and EDs, specifically with anorexia nervosa, supported by findings of overlapping phenotypes, e.g. elevated autism symptoms in participants with the ED and similarities in cognitive characteristics (Oldershaw et al., 2011; Westwood & Tchanturia, 2017). Anorexia nervosa is characterized by restrictive eating and significantly low body weight, fear of gaining weight, and a distorted perception of one’s body weight or shape (American Psychiatric Association, 2013). Despite the hypothesized link, research into the prevalence of clinical EDs including anorexia nervosa and bulimia nervosa in the autism population is still scarce, but current findings indicate that these conditions are more common among people diagnosed with autism and/or attention-deficit hyperactivity disorder (ADHD) than in the general population (Karjalainen et al., 2016; Råstam et al., 2013; Sedgewick et al., 2020). Also, the female:male ratio of EDs in people with neurodevelopmental conditions might not be as skewed towards females as in the general population (Karjalainen et al., 2016). Importantly, findings in the general population indicate that selective eating in childhood predicts anorexia nervosa symptoms in adolescence (Marchi & Cohen, 1990), suggesting that assessment and treatment of common eating problems in autism could be relevant for preventing the development of clinical EDs.

Studies show that eating problems are present also in other conditions commonly co-occurring with autism, which are therefore relevant to assess and account for in studies of the link between autism and eating problems to reduce confounding. ADHD and intellectual disability (ID) are found in around 28% and 30% of the autism population (Lai et al., 2019; Lyall et al., 2017), and have both been linked to eating problems, e.g. pica (eating substances that are considered inedible) is found in ID (Gravestock, 2000) and both ID and ADHD show increased selective eating (Gal et al., 2011; Smith et al., 2017). ADHD in particular has also been linked to EDs (Kaisari et al., 2017; Karjalainen et al., 2016), where increased impulsivity is associated to symptoms of bulimia nervosa (Kaisari et al., 2017), and co-occurring hyperactivity in autism has been related to lower body mass index (BMI) (Bölte et al., 2002). In addition, anxiety and depression disorders are also common in autism (Lai et al., 2019), and are associated to elevated ED symptoms (Fursland & Watson, 2014; Meng & D’Arcy, 2015).

## Gender and Eating Problems in Autism

Despite long-standing knowledge that females in the general population are at higher risk than males for developing EDs, including anorexia nervosa and bulimia nervosa (American Psychiatric Association, 2013; Bulik et al., 2007; Qian et al., 2013), and more frequently display behaviors associated with EDs (Solmi et al., 2021), little is known regarding gender differences in broader eating problems in the autism population. (Note: as behavioral differences between males and females might reflect both biological and sociocultural influences (Kreiser & White, 2014), which are difficult to disentangle (Lai et al., 2015), our study will use the term gender while acknowledging that both biological and sociocultural processes might be involved in shaping behavior). Gender-related findings in the autism phenotype, such as more pronounced sensory sensitivities among autistic females compared to autistic males (Lai et al., 2011), points to the possibility that eating behaviors might present differently in autistic females and males (Spek et al., 2020). Correspondingly, recent studies suggest that gender might influence eating problems in autism. For example, Van’t Hof et al. (2020) found that autistic traits and autism diagnosis predicted emotional undereating, i.e. eating less in response to emotional arousal such as being worried or angry, in girls but not boys. A recent study by Spek et al. (2020) investigated gender differences using self-ratings from neurotypical and autistic adults on the SWedish Eating Assessment for Autism spectrum disorders (SWEAA) questionnaire, which was developed to assess domains of common eating problems in autism (Karlsson et al., 2013). The study by Spek et al. found that autistic women reported more issues with purchase of food (e.g. only buying food from one brand) and sensory sensitivity associated with eating, while autistic men reported more motor problems in mealtimes (e.g. spilling when eating), when these groups were compared. When compared to neurotypical women, autistic women scored higher on eight of the ten SWEAA subscales reflecting, for instance, more problems with sensory sensitivity, selective eating, and symptoms of EDs. Importantly, autistic men also scored higher than neurotypical men on five of the subscales, including selective eating and inflexibility in mealtimes, indicating that challenges related to eating are present in adulthood among both women and men on the autism spectrum. While this study examined gender differences in a sample of adults with and without autism, it did not investigate the influence of gender on the relation between dimensional autistic traits and eating problems. Also, the few previous studies exploring gender differences in this area have not assessed and adjusted for other neurodevelopmental and psychiatric conditions that might confound the relation between autism and eating behaviors. Therefore, the current study included thorough assessments of ADHD, internalizing conditions (anxiety and depression disorders) and intelligence level to adjust for these potentially confounding variables. In addition, our study utilized a co-twin control design to adjust for a large number of unmeasured confounders, including genetics and shared environment, allowing investigation of influence from genetic and environmental factors on the link between autism and eating problems.

## Genetic and Environmental Underpinnings of Eating Problems

To the best of our knowledge, the underpinnings of the association between autism and eating problems, including selective eating, food neophobia, and food-related sensory issues, have not been investigated in previous research. However, twin-studies have helped to shed light on the etiology of eating problems and EDs in the general population. Food neophobia in the general population has been shown to be highly heritable, one twin-study reported a heritability estimate of 78% in 8–11 year old children, where the remaining variance was explained by non-shared environment, i.e. environmental factors that make twins in a pair different (Cooke et al., 2007). Similarly, substantial heritability has been found for both food neophobia and food selectivity in 16-month-old twins (Smith et al., 2017). For selective eating in particular, shared environment, referring to environmental factors that make twins in a pair more similar, was found to also play a role. Such influences could involve aspects of the family environment that are shared by both twins, such as how parents model eating behaviors (Cruwys et al., 2015; Smith et al., 2017). Regarding EDs, while it was previously believed that anorexia nervosa was mainly caused by sociocultural factors, family- and twin studies have highlighted the importance of genetic influence on the condition (Bulik et al., 2007). Accordingly, a Swedish twin-study reported that symptoms of EDs among children in the general population were accounted for by equal parts of genetic and non-shared environmental factors (Råstam et al., 2013). Although the underpinnings of the relation between autism and eating problems are not fully understood, it has been hypothesized to be explained by a common vulnerability to psychopathology and/or parent responses to the child in mealtimes (van ’t Hof et al., 2020), where findings from the general population suggest that pressuring a selectively eating child to eat might increase later food selectivity (Jansen et al., 2017). Family studies have also suggested a familial link between autism and anorexia nervosa, as the prevalence of autism is increased among relatives to individuals with anorexia nervosa (Koch et al., 2015). However, unlike twin-studies this type of family design cannot tease apart genetic and environmental influences. In the twin-study conducted by Råstam et al. (2013), cross-twin cross-trait correlations were calculated to assess if common genetic and environmental factors explain the phenotypic correlation between autistic traits and ED symptoms (failure to gain weight and fear of gaining weight), and the results suggested that the shared variance between the traits was explained by shared environment. While this study did explore the link between autistic traits and ED symptoms, it did not assess other common aspects of problematic eating in autism.

In summary, a body of research shows that autism is linked to problematic eating and suggests that this association also extend to levels of autistic traits in the general population (Sharp et al., 2013; van ’t Hof et al., 2020; Wallace et al., 2018). Growing evidence indicate that gender influences the extent and type of eating problems in autism, yet more research is needed regarding the nature of eating problems exhibited by autistic females compared to males. Furthermore, research on gender-related differences in autism has rarely investigated the association of both dimensional autistic traits and categorical autism diagnosis with eating problems. Moreover, research in this field has rarely taken commonly co-occurring conditions or potential moderators into account that might influence eating behavior such as ADHD, internalizing conditions, and intellectual abilities.

In this study, we assessed different aspects of eating problems in a sample of adolescent and adult twins enriched for autism and other neurodevelopmental conditions. The aims of this study were threefold. First, to examine the relationship between autism (dimensional and categorical) and eating problems across the sample of twins, investigating both the overall extent and presence of specific eating problems, while adjusting for covariates (gender, age, ADHD, internalizing conditions, and IQ). Second, to investigate potential gender-specific associations between autism and eating problems. Finally, to explore the influence of shared genetic and environmental factors on the relation between autism and eating problems using a co-twin control design and within-pair analysis. Based on previous research and clinical experience, we predicted that autistic traits and autism diagnosis would be associated with increased eating problems, and we predicted that these associations would be found particularly in females. We did not set up a specific hypothesis for the third aim as previous research related to this aim is scarce.

## Methods

### Participants

The sample included 192 twins (55% females, mean age: 21.1, range 15–33 years), comprising 63 monozygotic (MZ) and 33 dizygotic (DZ) twin pairs, who participated in the Roots of Autism and ADHD Twin Study in Sweden (RATSS) (Bölte et al., 2014). Of these participants, 28 fulfilled criteria for an autism diagnosis (15 females and 13 males), 28 for ADHD, and six for mild ID. A proportion of the participants had more than one of the above conditions (i.e. eight participants with autism had co-occurring ADHD and were thus included in both diagnostic groups). Two of the participants fulfilled criteria for an ED, both were diagnosed with bulimia nervosa (one of these participants had ADHD, none were diagnosed with autism). In the full sample 46 participants fulfilled criteria for an internalizing condition (e.g. social anxiety disorder, specific phobia, unspecified anxiety disorder, and major depression). The sample included 16 twin pairs discordant for autism diagnosis (7 MZ pairs and 9 DZ), and 59 pairs discordant for autistic traits (32 MZ and 27 DZ pairs), i.e. showing a minimum of seven points intra-pair difference on Social Responsiveness Scale-2 (SRS-2) total raw score, reflecting the standard error of measurement (Constantino & Gruber, 2012). Sample characteristics are summarized in Table 1.

**Table 1:** Sample characteristics

	Total n = 192	Males n = 86	Females n = 106	Autism n = 28
Age, M (SD), Range	21.06 (4.96), 15–33	19.86 (4.40), 15–31	22.04 (5.19), 15–33	20.29 (4.40), 15–31
Autism	28	13	15	28
ADHD	28	17	11	8
Intellectual disability	6	4	2	2
Internalizing conditions	46	16	30	7
IQ, M (SD), Range	100.00 (16.13) 62–142	100.37 (15.88) 62–131	99.70 (16.40) 63–142	92.86 (21.22) 63–142
SWEAA total, M (SD)	12.83 (8.62)	13.02 (7.56)	12.67 (9.44)	19.68 (9.08)
SRS-2 total raw, M (SD)	30.88 (28.07)	32.99 (27.99)	29.17 (28.15)	77.25 (27.18)

*M* mean, *SD* standard deviation, *ADHD* attention deficit hyperactivity disorder, *IQ* intelligence quotient, *SWEAA* Swedish Eating Assessment for Autism spectrum disorders, *SRS-2* Social Responsiveness Scale–Second Edition

Twins were mainly recruited from the population-based studies Child and Adolescent Twin Study in Sweden (CATSS) (Anckarsäter et al., 2011) and the Young Adult Twins in Sweden Study (YATSS) (Zagai et al., 2019), prioritizing twin pairs where one or both twins show elevated scores on the autism- or ADHD-screening scales. In addition, twin pairs with no indications of neurodevelopmental conditions were recruited as controls. Diagnoses in RATSS are determined through comprehensive assessments during a 3-day visit at a clinical research unit, including cognitive testing, clinical interviews, and self- and parent-report questionnaires. Twin zygosity was determined on a panel of 48 single nucleotide polymorphisms for 83 pairs (Hannelius et al., 2007), or by parent-report on a 4-item zygosity questionnaire (for 13 pairs).

**Fig. 1:**
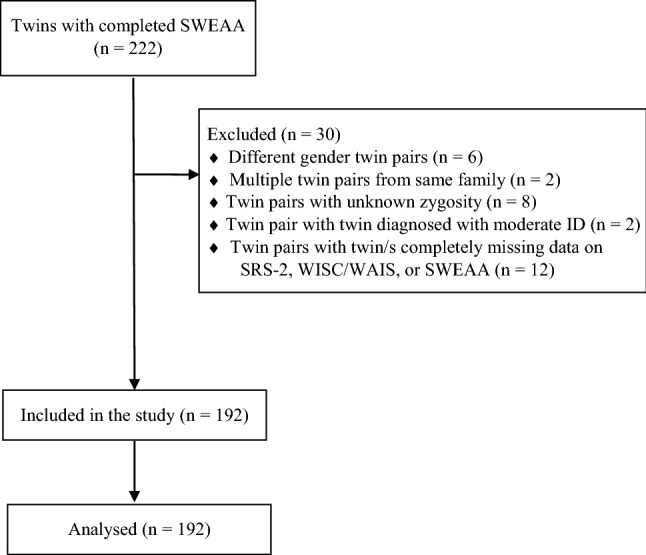
Participant flow chart showing exclusion reasons and twins included in the analyses

In the total RATSS sample, a total of 222 participants had completed the SWEAA. Twin pairs that were of different gender were excluded (n = 6). From one family two twin-pairs had participated and one pair was thus excluded (n = 2). Furthermore, we excluded twin pairs where one twin had not completed the SWEAA (n = 2), or where one or both twins had completely missing data on SRS-2 (n = 6) or the Wechsler Intelligence Scales (n = 4). Finally, twins where zygosity had not yet been determined (n = 8), or where one twin had a moderate ID (n = 2) were excluded, resulting in the final sample of 192 participants. Figure 1 shows excluded twin pairs and the final sample included in the analyses.

### Measures

Twins participated in comprehensive assessments conducted by experienced clinicians. Consensus research DSM-5 diagnosis of autism was supported by the Autism Diagnostic Observation Schedule Generic or 2^nd^ edition (ADOS-G or ADOS-2) (Gotham et al., 2007; Lord et al., 2012) and Autism Diagnostic Interview – Revised (ADI-R) (Rutter et al., 2003). Autistic traits were measured by parent-report on the child or adult version of the SRS-2 (Constantino & Gruber, 2012), using total raw scores as recommended for research purposes (Constantino, 2005). A small proportion of the participants (n = 20) had up to four missing items in SRS-2 (13 had one missing item and 7 more than one). Values for these items were imputed in accordance with the SRS-2 manual (Constantino & Gruber, 2012).

Diagnoses of ADHD, other neurodevelopmental and psychiatric conditions were based on information from a multitude of sources, including medical history, parent interview on the Kiddie Schedule for Affective Disorders and Schizophrenia (K-SADS) (Kaufman et al., 1997) for twins younger than 18 years, parent and twin interview on the Diagnostic Interview for ADHD in adults (Kooij, 2010) and twin interview on the Structured Clinical Interview for DSM-IV (SCID, axis I) for twins who were 18 years or older. The General Ability Index (GAI) from the Wechsler Intelligence Scales for Children – Fourth edition (WISC-IV) or the Wechsler Adult Intelligence Scale – Fourth Edition (WAIS-IV) (Wechsler et al., 2008) were used to measure IQ, and to endorse diagnosis of ID.

Eating problems common in autism were assessed using self-ratings on the SWEAA, which is a questionnaire developed based on a literature review of eating problems among autistic adolescents and adults (Råstam, 2008) as well as the authors’ clinical experience (Karlsson et al., 2013). The questionnaire consists of 60 items rated on a 5-point Likert scale ranging from “never” to “always” (scored 0–4) (Karjalainen, 2016; Karlsson et al., 2013), encompassing different domains of eating behaviors, such as selective eating, sensory sensitivity around food, and rituals and routines at mealtimes. The SWEAA is divided into eight subscales: *A. Perception* (11 items), *B. Motor control* (7 items), *C. Purchase of food* (3 items), *D. Eating behavior* (6 items), *E. Mealtime surroundings* (11 items), *F. Social situation at mealtime* (10 items), *G. Other behavior associated with disturbed eating* (8 items), and *H. Hunger/satiety* (2 items) and two single-item subscales, *I. Simultaneous capacity* and *J. Pica*. See Table 2 for a description of the subscales and example items. Subscale G pertain to clinical EDs, and include fasting, dieting, and purging (e.g. inducing vomiting after meals). Using the SWEAA to measure eating problems therefore yields a broad operationalization of the phenomena incorporating eating behavior associated with autism (e.g. selective eating) as well as symptoms of clinical EDs. SWEAA has been shown to discriminate well between autistic and neurotypical adults (Karlsson et al., 2013; Spek et al., 2020), and has demonstrated good internal consistency, as well as test–retest reliability (Karlsson et al., 2013). In line with the recommendations of the authors of the questionnaire, we excluded items that were not included the final version of SWEAA, yielding a total of 60 items (Karlsson et al., 2013). Thus, the analyses in our study use the final version of the SWEAA that has been validated, and used in previous studies (Demartini et al., 2021; Karjalainen et al., 2019; Karlsson et al., 2013; Spek et al., 2020). While different scoring procedures have been used for SWEAA, we used the scoring recommended by the authors (Karjalainen, 2016; Karlsson et al., 2013), i.e. a mean was calculated for the items within a subscale which was subsequently multiplied with 25 yielding a score ranging from 0 to 100 for each subscale, with a higher score indicating more eating problems. The same scoring procedure using all 60 items was used to calculate the SWEAA total score. Eleven participants in our final sample had a maximum of two missing items in SWEAA. For these, subscale scores were calculated based on the available items, i.e. if an item was missing from the *Perception* subscale which consists of seven items, the mean of the available six items was used.

**Table 2:** Descriptions of SWEAA subscales and example items

Subscales	Example items
*Perception*: reflects sensory input related to food, such as smell, taste, texture or sound	I am oversensitive to certain flavors
*Motor control*: assesses different aspects of movement, such as chewing, spilling or table manners	I find it difficult to chew
*Purchase of food*: concerns the control of purchases; for instance, brands or type of groceries	My food must be of a certain brand
*Eating behavior*: indicates selectivity in eating, such as certain colors, limited repertoire or trying new foods	I only eat a limited menu, maximum of 10 dishes
*Mealtime surroundings*: reflects routines around mealtimes; for example, where to eat or how cutlery is placed	I have certain rituals around mealtimes
*Social situation at mealtime*: assesses the situation in relation to others at mealtime, such as adapting own behavior to that of others or enjoying company during a meal	I look down at my food most of the time during a meal
*Other behaviors associated with disturbed eating*: questions of traditional eating disorders, such as fasting, purging or dieting	I induce vomiting after meals
*Hunger/Satiety*: measures if the individual can feel when hungry or full	I feel when I am hungry
*Simultaneous capacity*: indicates whether the individual finds it hard to do two things simultaneously during a meal	I find it difficult to do two things simultaneously during a meal, e.g. chewing and cutting the food
*Pica*: measures whether the respondent eats inedible things, such as soil or mortar	I eat things that others consider inedible (e.g. mortar or soil)

From Karjalainen et al. (2019)

### Statistical Analyses

All analyses were conducted in R within the Generalized Estimating Equations (GEE) framework with doubly robust standard errors (drgee package) (Zetterqvist & Sjölander, 2015), which does not make assumptions regarding the distribution of the data. This approach allows applying regression models in a twin sample with standard errors that account for the clustering of the data (i.e. between twins in a pair). In addition, it allows estimating within-pair associations, i.e. whether the twin who has higher autistic traits in a twin pair also display more eating problems compared to their co-twin. Conclusions regarding genetic and environmental influences on an association can be drawn by comparing within-pair associations between monozygotic and dizygotic groups and to associations across the sample (McGue et al., 2010). The GEE framework has been employed in previous twin studies conducted by our group (e.g. Isaksson et al., 2019; Neufeld et al., 2021). We report the unstandardized estimates, presenting the change in SWEAA score associated with one point increase on the SRS-2, unless we explicitly refer to standardized estimates. The analyses are described in detail below.

#### Across-Individuals Analyses

The association between autism and eating problems was examined across individuals using linear regression models with cluster-robust standard errors. For our first aim, to examine the link between autism and eating problems, an unadjusted model was conducted using autistic traits as exposure and total eating problems score as outcome. Subsequently, a model adjusted for gender, age, ADHD, internalizing conditions, and IQ was run. To investigate the link to specific eating problems, separate models examining the association between autistic traits and the SWEAA subscales were conducted, using Bonferroni correction for multiple comparisons (10 separate models: p-value threshold = 0.05/10 = 0.005, i.e. p-values below 0.005 were considered significant). The subscale models were adjusted for the variables described above. We conducted follow-up analyses using autism diagnosis as exposure for SWEAA scores that showed a significant association with autistic traits to confirm the clinical relevance of the results. To address our second aim, the adjusted models included the interaction term autism (traits or diagnosis) by gender, to allow investigation of gender-specific effects of autistic traits and autism diagnosis on eating problems. Furthermore, follow-up analyses stratified by gender were conducted for models showing a significant interaction between autism and gender. As all follow-up analyses were conducted to further explore and validate the associations between autistic traits and eating problems, these models were not Bonferroni corrected, i.e. the significance level was set to p = 0.05.

#### Within-Pair Analyses

For our third aim, within-pair analyses were conducted using conditional linear regressions in order to investigate the influence of genetics, shared environment and non-shared environment on the association between autistic traits and total eating problems. These analyses implicitly control for all variables shared within twin pairs, including shared environment and genes (100% of segregating genes for monozygotic [MZ] pairs and on average 50% for dizygotic [DZ] pairs). The within-pair analyses were only adjusted for ADHD, internalizing conditions, and IQ, as age and gender are implicitly controlled for. A within-pair analysis was first run in the entire sample (96 twin pairs), and subsequently split in MZ- and DZ-twin pairs. A weaker association in MZ-pairs compared to DZ-pairs indicates a genetic influence on the association, while a lost association within both MZ- and DZ-pairs would indicate a shared environmental influence. An association that remains within MZ-pairs is consistent with autistic traits having a causal effect on eating problems, but could also indicate a common influence from non-shared environmental factors.

#### Power Analysis

Power calculations for this study are approximal, due to the sample consisting of twins and the sampling approach. More specifically, participants were not selected randomly, rather the sampling aimed to include a high proportion of pairs discordant and concordant for autism and ADHD because these are most informative for studying these conditions. A post-hoc power analysis using G*power 3.1 (Faul et al., 2009) yielded that our sample size of 192 participants was sufficient to detect a medium effect size (minimum effect of f^2^ = 0.073) with a power of 0.80 given an α of 0.05, using a multiple regression with six predictors in across-individuals analyses.

## Results

### Across-Individuals Analyses

#### Association Between Autism and Total Eating Problems

Across-individuals results for total eating problems in autism are presented in detail in Table 3. Autistic traits were associated with an increase in total eating problems, and the association remained when adjusting for gender, age, ADHD, internalizing conditions, and IQ. An increase of one point on the SRS-2 predicted an increase of 0.17 points on the SWEAA total score, in the adjusted model. Correspondingly, autism diagnosis also predicted more eating problems in both the unadjusted and adjusted model, where autism diagnosis was associated with an increase of 9.94 points on the SWEAA total score in the latter. The standardized estimates show that one standard deviation increase in autistic traits was associated with an increase of 0.56 standard deviations in SWEAA total score, and autism diagnosis was associated with an average increase of 1.15 standard deviations in SWEAA total score, in the adjusted models. In the autistic traits model, male gender and internalizing conditions were associated with increased total eating problems. In the model using autism diagnosis as exposure, internalizing conditions and ADHD diagnosis were associated with increased total eating problems, while higher age was associated with a lower total score.

**Table 3:** Across-individuals association between dimensional/categorical autism and total eating problems

	Unadjusted model			Adjusted model		
	b (95% CI)	SE	p	b (95% CI)	SE	P
*Autistic traits*	**0.14 (0.10 to 0.19)**	**0.02**	** < 0.001**	**0.17 (0.11 to 0.24)**	**0.03**	** < 0.001**
Gender				**3.49 (0.33 to 6.65)**	**1.61**	**0.030**
Age				− 0.19 (− 0.41 to 0.04)	0.11	0.101
ADHD				2.67 (− 0.97 to 6.30)	1.85	0.151
Internalizing conditions				**3.83 (0.69 to 6.97)**	**1.60**	**0.017**
IQ				0.005 (− 0.08 to 0.09)	0.04	0.912
Autistic traits x Gender				**− 0.12 (− 0.20 to − 0.05)**	**0.04**	** < 0.001**
*Autism*	**8.02 (4.35 to 11.70)**	**1.87**	** < 0.001**	**9.94 (5.07 to 14.81)**	**2.48**	** < 0.001**
Gender				0.60 (-2.18 to 3.37)	1.42	0.673
Age				**− 0.34 (− 0.57 to − 0.10)**	**0.12**	**0.005**
ADHD				**3.98 (0.28 to 7.68)**	**1.89**	**0.035**
Internalizing conditions				**4.51 (1.25 to 7.76)**	**1.66**	**0.007**
IQ				− 0.02 (− 0.10 to 0.06)	0.04	0.663
Autism x Gender				− 6.66 (− 14.48 to 1.16)	3.99	0.095

*b* regression coefficient, *CI* confidence interval, *SE* standard error. Significant results in bold (p < 0.05)

#### Association Between Autism and Specific Eating Problems

See Table 4 for across-individuals associations between autistic traits and SWEAA subscales. In these adjusted models, autistic traits were associated with the subscales *Perception*, *Eating behavior*, *Mealtime surroundings*, *Social situation at mealtime*, and *Simultaneous capacity*. The five subscales showing an association with autistic traits were followed-up in regressions using autism diagnosis as the exposure, which largely confirmed the findings, except for the subscale *Simultaneous capacity* where no association with autism diagnosis was found (see Table 5). The standardized estimates showed that having a diagnosis of autism predicted approximately one standard deviation increase on the subscales *Perception*, *Eating behavior*, *Mealtime surroundings*, and *Social situation at mealtime* (range 0.94 *–* 1.14, with *Perception* showing the strongest association)*.*

**Table 4:** Across-individuals association between autistic traits and SWEAA subscales

	Autistic traits	Gender	Age	ADHD	Internalizing conditions	IQ	Autistic traits x Gender
	b/SE/p	b/SE/p	b/SE/p	b/SE/p	b/SE/p	b/SE/p	b/SE/p
A. Perception	**0.23/0.06/ < 0.001**	0.37/2.35/ 0.875	− 0.33/0.19/0.086	4.53/3.09/0.143	1.95/2.11/0.355	0.09/0.07/0.228	− 0.15/0.07/0.038
B. Motor control	0.08/0.03/0.021	3.57/2.29/0.119	− 0.18/0.15/0.223	0.82/2.60/0.753	**5.66/1.99/ < 0.005**	0.02/0.04/0.679	− 0.06/0.06/0.331
C. Purchase of food	0.06/0.07/0.377	2.91/3.85/0.451	0.87/0.35/0.013	3.90/3.51/0.267	2.35/3.55/0.508	0.06/0.09/0.466	− 0.14/0.08/0.060
D. Eating behavior	**0.24/0.06/ < 0.001**	7.25/3.22/0.024	− 0.05/0.22/0.805	2.08/3.25/0.521	4.69/3.03/0.122	0.04/0.07/0.621	− 0.15/0.09/0.098
E. Mealtime surroundings	**0.25/0.06/ < 0.001**	5.34/2.15/0.013	− 0.34/0.16/0.032	3.69/2.66/0.165	5.32/2.26/0.019	− 0.06/0.05/0.189	**− 0.27/0.06/ < 0.001**
F. Social situation at mealtime	**0.24/0.05/ < 0.001**	**8.54/2.60/0.001**	− 0.29/0.17/0.078	− 1.49/2.92/0.610	3.89/2.14/0.069	− 0.05/0.06/0.425	− 0.05/0.07/0.509
G. Other behavior associated with disturbed eating	0.004/0.03/0.889	− 1.88/1.82/0.302	− 0.07/0.11/0.499	2.36/2.02/0.243	1.57/1.52/0.301	− 0.07/0.04/0.076	− 0.01/0.03/0.648
H. Hunger/Satiety	0.22/0.08/0.010	− 0.60/4.35/0.890	− 0.55/0.33/0.093	14.44/6.66/0.030	10.16/4.03/0.012	0.24/0.10/0.019	− 0.20/0.10/0.047
I. Simultaneous capacity	**0.13/0.04/0.001**	1.90/1.60/0.234	− 0.20/0.14/0.155	7.71/4.34/0.075	3.00/2.28/0.188	0.02/0.06/0.749	− 0.13/ 0.06/0.020
J. Pica	0.004/0.01/0.750	− 1.05/1.81/0.560	− 0.02/0.06/0.687	− 1.45/1.80/0.420	0.06/0.91/0.945	− 0.02/0.01/0.211	0.06/0.06/0.338

*b* regression coefficient, *SE* standard error. Significant results in bold. Bonferroni corrected significance level (p < 0.005)

**Table 5:** Across-individuals associations between autism diagnosis and SWEAA subscales

	Autism	Gender	Age	ADHD	Internalizing conditions	IQ	Autism x Gender
	b/SE/p	b/SE/p	b/SE/p	b/SE/p	b/SE/p	b/SE/p	b/SE/p
A. Perception	**15.04/5.22/0.004**	− 3.42/1.93/0.076	**− 0.54/0.19/0.006**	6.06/3.14/0.054	2.83/2.10/0.177	0.07/0.07 /0.354	− 6.58/7.25/0.364
D. Eating behavior	**13.30/5.22/0.011**	3.55/2.55/0.165	− 0.28/0.23/0.230	4.09/3.32/0.218	5.57/3.03/0.066	0.004/0.07/0.955	-5.87/8.32/0.480
E. Mealtime surroundings	**13.57/3.47/ < 0.001**	− 0.60/1.85/0.744	**− 0.53/0.17/0.002**	5.04/2.73/0.064	**6.34/2.34/0.007**	**− 0.09/0.05/0.049**	**− 17.46/5.22/ < 0.001**
F. Social situation at mealtime	**14.64/3.61/ < 0.001**	**7.60/2.04/ < 0.001**	**− 0.53/0.15/ < 0.001**	1.21/2.75/0.659	**4.92/2.14/0.022**	− 0.10/0.06/0.119	− 3.43/5.47/0.530
I. Simultaneous capacity	5.86/3.60/0.104	− 1.86/1.98/0.346	**− 0.31/0.15/0.031**	8.38/4.42/0.058	3.33/2.29/0.146	0.01/0.06 /0.878	− 3.08/5.32/0.563

b: regression coefficient; SE: standard error. Significant results in bold (p < 0.05)

Regarding covariates, male gender was associated with a higher score on the subscale *Social situation at mealtime* in both the model using autistic traits as exposure and the autism diagnosis model. Having at least one internalizing condition was associated with more self-reported problems with *motor control* when autistic traits were used as exposure, and higher scores on *Mealtime surroundings* and *Social situation at mealtime* in the autism diagnosis models. In the diagnosis models only, higher age was associated with reduced scores in subscales *Perception*, *Mealtime surroundings*, *Social situation at the mealtime*, and *simultaneous capacity*. Also, in the autism diagnosis model, higher IQ was associated with lower scores on *Mealtime surroundings*.

#### Interaction Effects

**Fig. 2:**
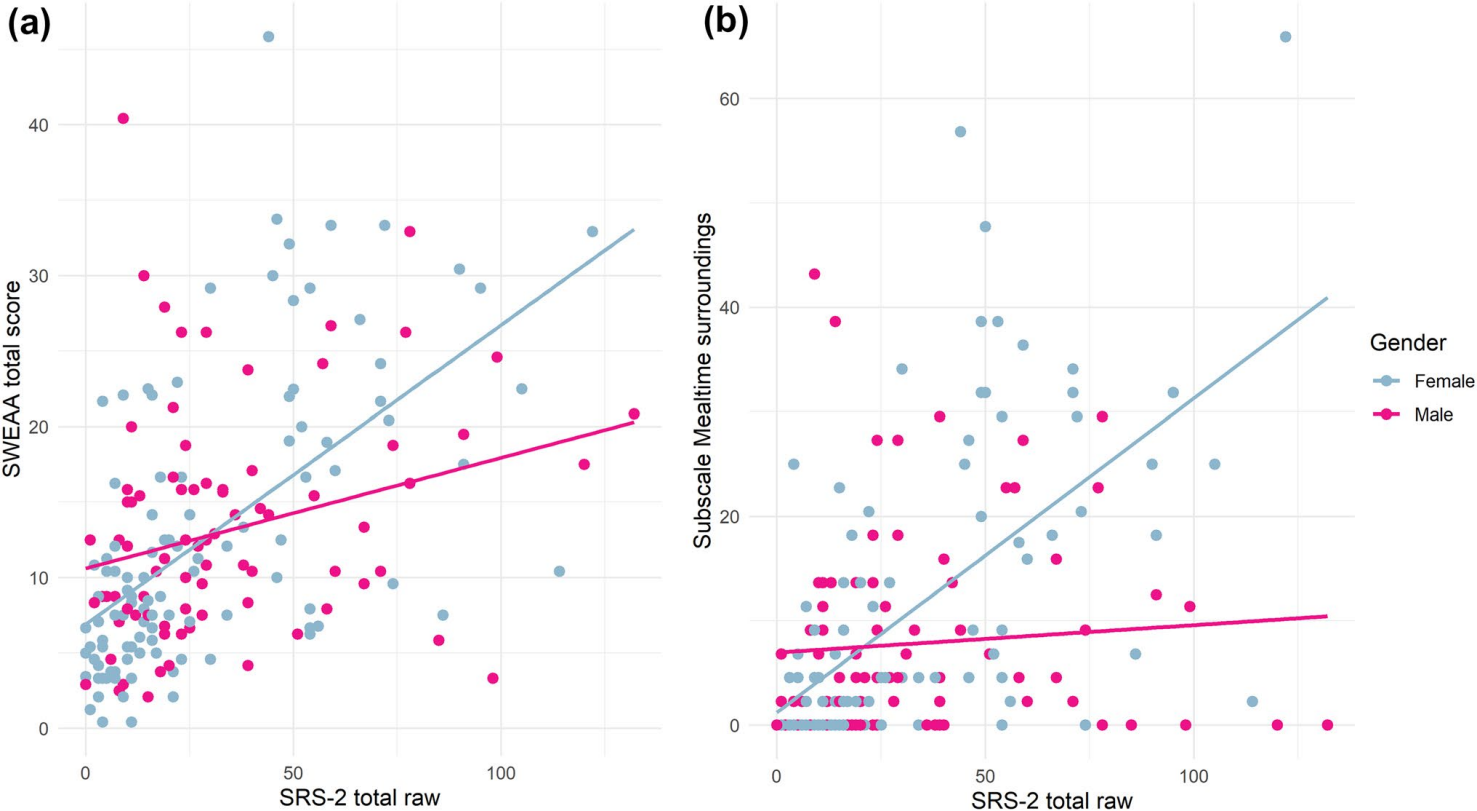
Interaction effects between autistic traits and gender in across-individuals analyses. The regression lines show the unadjusted association between autistic traits and either SWEAA total score (**a**) or score on the Mealtime surroundings subscale (**b**) for females and males separately

An interaction effect between autistic traits and gender was found for total eating problems (see Table 3). Follow-up analyses showed a stronger association between autistic traits and total eating problems for females (b = 0.14, 95% CI 0.07 to 0.21, p < 0.001) than for males (b = 0.06, 95% CI 0.01 to 0.11, p = 0.019), adjusting for age, ADHD, internalizing conditions and IQ. For females, having a diagnosis of ADHD or an internalizing condition was associated with an increase in total eating problems, and higher age with decreased eating problems, while no covariates were significant in males. An interaction effect was also found for the *Mealtime surroundings* subscale (see Table 4), where an association between autistic traits and increased self-reported problems was found specifically for females (b = 0.20, 95% CI 0.09 to 0.31, p < 0.001), while no association was found for males. The same pattern was found for autism diagnosis, which was associated with more self-reported problems in *Mealtime surroundings* for females (b = 10.98, 95% CI 4.25 to 17.71, p = 0.001), but not males. Figure 2 shows the associations between autistic traits and total eating problems as well as *Mealtime surroundings* for females and males respectively. For further exploration of this interaction effect, *Mealtime surroundings* was split into items focusing on eating in social contexts (six items including difficulties eating in school or with friends) and non-social items focusing on rituals and rigidity in mealtimes (five items). An interaction effect was only found for the cluster of social items, where autistic traits and autism diagnosis predicted increased scores for females but not males. These analyses are described in detail in the Supplementary Material. Supplementary Table 1 provides details on the follow-up analyses for females and males separately.

### Within-Pair Analyses

#### Association Between Autism and Total Eating Problems

**Fig. 3:**
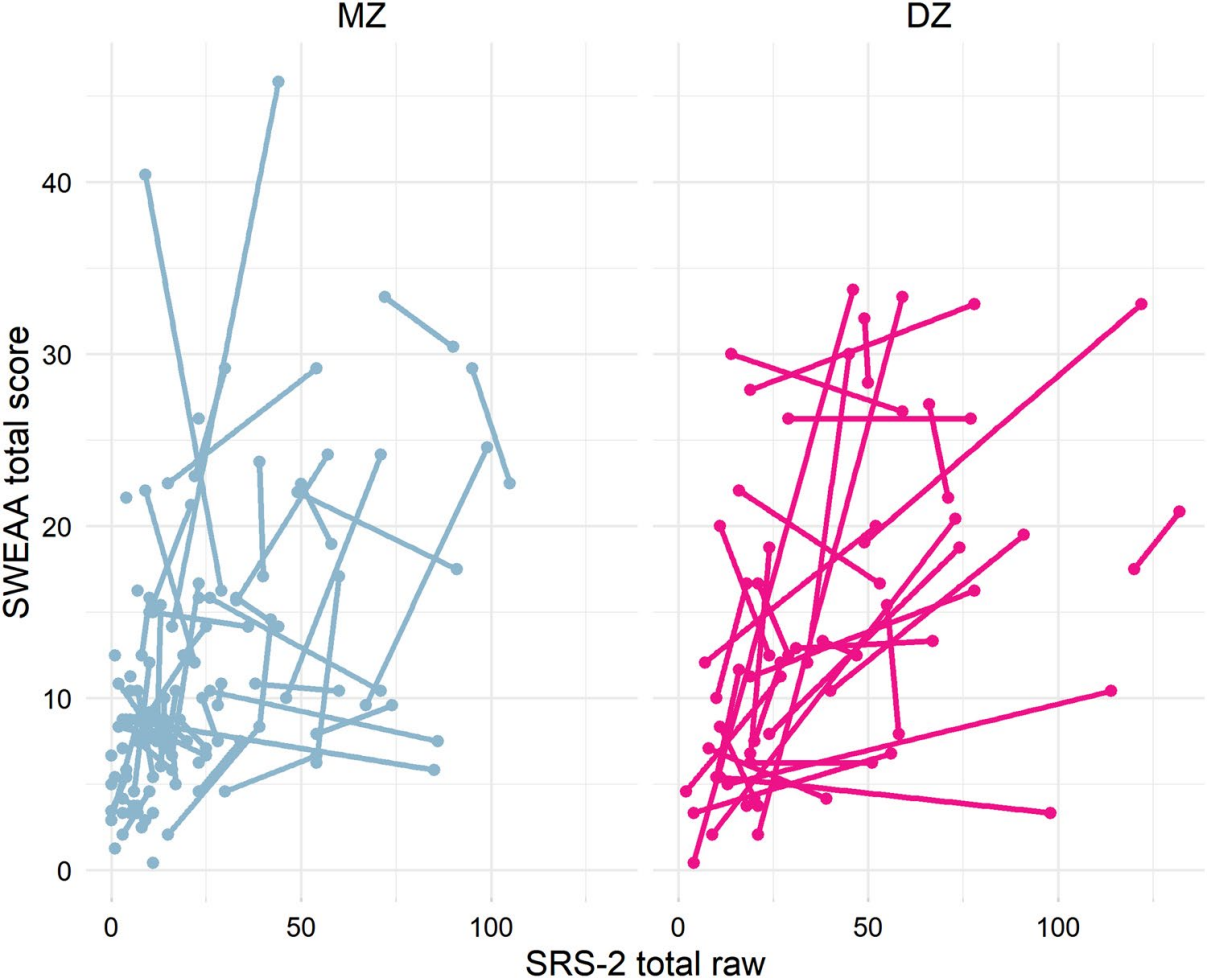
Within-pair associations between autistic traits and total eating problems in MZ- and DZ-pairs. The twins in each pair are shown connected with a line

In the within-pair model, autistic traits were associated with total eating problems in both the unadjusted model (b = 0.10, 95% CI   0.03 to 0.17, p = 0.003), and in the adjusted model (b = 0.09, 95% CI   0.02 to 0.16, p = 0.008). The adjusted association was significant within DZ-pairs (b = 0.11, 95% CI   0.04 to 0.18, p = 0.003) but not within MZ-pairs (b = 0.04, 95% CI  − 0.08 to 0.17, p = 0.484). ADHD, internalizing conditions and IQ were not associated with eating problems in the above within-pair models. Figure 3 visualizes the within-pair associations between autistic traits and total eating problems for MZ- and DZ-pairs. Restricting the sample to only the 16 twin-pairs categorically discordant for autism (7 MZ and 9 DZ), a similar pattern was found, showing a significant association between autism diagnosis and total eating problems within DZ-pairs (b = 5.56, 95% CI 1.15 to 9.97, p = 0.013) but not within MZ-pairs (b = 2.07, 95% CI − 1.25 to 5.40, p = 0.221). Internalizing conditions was negatively associated with eating problems in this model (b = − 5.22, 95% CI -8.83 to − 1.61, p = 0.005).

#### Association Between Autism and Specific Eating Problems

The SWEAA-subscales that showed associations with autistic traits across individuals were followed up in separate within-pair models. Within-pair associations for three of the SWEAA-subscales followed the same pattern as total eating problems, showing significant associations only in DZ-pairs (*Perception*, *Mealtime surroundings*, and *Social situation at mealtime*, see Supplementary Table 3). In contrast, the subscale *Eating behaviors* showed a significant association within both MZ- and DZ-pairs. For *Simultaneous capacity*, no association was found within MZ- or DZ-pairs.

## Discussion

In this co-twin control study, autistic traits were associated with an increase in total eating problems, and with specific aspects of eating problems, including sensory issues in mealtimes, selective eating, inflexible routines and rituals at mealtimes, difficulties adapting eating behavior to others and problems with simultaneous capacity. The results were also largely supported using autism diagnosis as the exposure, indicating their clinical relevance. Interaction effects showed that autistic traits predicted elevated overall eating problems especially in females. Furthermore, a link between (dimensional and categorical) autism and self-reported difficulties with eating in social situations outside of the home was found specifically among females. The interaction effects suggest that autism and eating behavior are differentially associated among females and males. In the within-pair analyses, the association between autism and total eating problems remained significant in DZ-pairs but was lost within MZ-pairs, which might suggest a genetic influence on the association. However, the interpretation of these results should be made with caution, as confidence intervals of MZ- and DZ-twins were overlapping.

In line with our hypothesis, the results corroborate that eating and mealtime challenges are common among autistic adolescents and adults (Karlsson et al., 2013; Råstam, 2008; Spek et al., 2020). Corresponding to results from the SWEAA validation study (Karlsson et al., 2013), we found that both autistic traits and autism diagnosis were associated with increased self-reported problems with adapting to company during meals (*Social situation at the mealtime*) and, particularly among female participants, with inflexible routines and rituals in mealtimes (*Mealtime surroundings*). While autistic participants in the SWEAA validation study self-reported more difficulties with *Simultaneous capacity*, our data only supported an association between autistic traits and scores on this subscale. Unlike the validation study, we also saw associations between autism and subscales encompassing sensory issues (*Perception*) and selective eating (*Eating behavior*), reflecting recent findings in the study by Spek et al. (2020) where both autistic men and women showed elevated selective eating compared to neurotypical men and women, and autistic women reported more sensory issues in mealtimes compared to neurotypical women.

We did not find a significant association between autistic traits and ED symptoms measured by the SWEAA subscale *Other behavior associated with disturbed eating*, including fasting, dieting, and purging. Autism and autistic traits have been linked to elevated ED symptoms in some studies (Kalyva, 2009; Karjalainen et al., 2016; Sedgewick et al., 2020), but not all (Karlsson et al., 2013; van ’t Hof et al., 2020). The lacking associations in our data should however be interpreted with caution, as the SWEAA subscale does not fully cover central ED symptoms such as fear of gaining weight and binge eating, and the authors of SWEAA note that items focusing on EDs were excluded in the validation process due to not discriminating between participants with autism and controls (Karjalainen et al., 2019). Potentially, including more items relevant to EDs in our study could have generated different results. Further research is needed to investigate the association between autism and ED symptoms.

Our findings also indicate that ADHD is related to an increase in total eating problems beyond autism diagnosis. This association was however not found in the autistic traits model, potentially reflecting that participants with ADHD also have elevated autistic traits (Bölte et al., 2018). Lower IQ did not appear to be related to problematic eating in the aspects measured by the SWEAA, but regarding inflexibility in mealtimes (*Mealtime surroundings*) participants with higher IQ displayed less problems, when autism diagnosis was used as exposure. Internalizing conditions were also related to a higher SWEAA total score, suggesting that certain eating problems associated with autism also occur in anxiety and depression conditions.

The gender-related associations found in our study add to the literature suggesting gender differences in eating behavior in autism (Spek et al., 2020; van ’t Hof et al., 2020). As the main effect of gender on total eating problems showed that males in general rather scored higher, our findings do not appear to reflect a typical gender difference in the general population but instead indicate that the link between autism and problems regarding food and mealtimes is particularly pronounced among females. Furthermore, our results suggest that the social aspect of eating problems might be particularly relevant for autistic females. The association between autism and the *Mealtime surroundings* subscale, specifically regarding social items, was found exclusively among females, suggesting that eating together with others is a particular challenge for autistic females. This might have detrimental effects in limiting opportunities of social interaction with peers in situations where one is expected to eat together with others, e.g. during lunch in school or at work, in cafés or restaurants. Potentially contributing factors were reported in a qualitative study including mainly autistic women, where participants described that they would avoid communal eating settings such as restaurants and school cafeterias due to sensory sensitivities concerning noise and smells (Kinnaird et al., 2019). Participants in the study by Kinnaird and colleagues also expressed that embarrassment over their own eating behaviors or difficulties would lead them to eat alone or only with close acquaintances. While the only previous study comparing self-reports on the SWEAA between autistic females and males did not find a significant difference between 30 autistic men and 36 autistic women on the *Mealtime surroundings* subscale, the difference between the groups was approaching significance (p = 0.058) (Spek et al., 2020), and the nonsignificant result might have reflected lack of power.

While autistic traits and autism were related to difficulties in eating with others outside of the home exclusively in females, a main effect of gender was found for the *Social situation at mealtime* subscale where male gender was associated to more problems in adapting behavior to others in mealtimes. However, these two subscales cover different areas of the social aspect of mealtimes. Where the items in *Mealtime surroundings* endorsed by autistic females in our sample reflects experiencing difficulties in eating with others (e.g. “I find it difficult to eat at school/workplace/activity centre or similar”), the items in *Social situation at mealtime* reflects challenges in adapting behavior to others (e.g. “I look down at my food most of the time during the meal”) and preference to eat alone.

The link between adolescent and adult autism and the various issues around eating and mealtimes suggested by our results is often not investigated in health care services encountering this population, increasing the risk of the eating problems seen in our study going unnoticed. For clinicians, our results emphasize the relevance of assessing eating problems in this group, but also in the wider population displaying elevated autistic traits but not diagnosed with autism. Our data further suggests that this might be of particular relevance when meeting females with elevated autistic traits. Further, the eating problems in people with high levels of autistic traits and/or diagnosed autism might not be in the form of anorexia or bulimia nervosa symptoms but rather concern other areas such as eating a limited diet or experiencing problems with sensory aspects of food or mealtimes, which psychiatry services might not be sufficiently familiar with. Individuals presenting with the eating problems described in our study might suffer from consequential detrimental effects on quality of life and functioning, including social participation, even if criteria for an ED are not fulfilled. Behavioral interventions for severe eating problems show promising results for adults in the general population (Thomas et al., 2021), and could potentially also be helpful for some of the eating issues described in our data. However, such interventions might need adaption for people with elevated autistic traits or autism, which is underlined by previous findings suggesting that standard ED treatments might not be as effective for people with elevated autistic traits (Westwood & Tchanturia, 2017).

## Underpinnings of the Link Between Autism and Eating Problems

In the within-pair analysis, the association between autistic traits and total eating problems was significant in DZ-pairs, who share on average 50% of their genes, but lost in MZ-pairs, who share 100% of their genes, despite a larger representation of MZ-pairs in our sample (63 MZ vs. 33 DZ pairs). Similarly, the association between autism diagnosis and overall eating problems was significant only among DZ-pairs when restricting the sample to the 16 pairs discordant for autism. Thus, the within-pair analyses might indicate a genetic influence on the association between autism and eating problems. While previous research indicates a substantial genetic influence in both autism (Tick et al., 2016) and in eating problems such as food neophobia and selective eating (Cooke et al., 2007; Smith et al., 2017), our results point towards a potential influence of common genetic factors on both variables. This could reflect a genetic influence on sensory sensitivity, which is included in the core RRBI domain in autism and might function as a basis for a variety of eating problems. Our within-pair analyses pointed towards a potential genetic influence on the relationship between autistic traits and sensory issues in mealtimes (the *Perception* subscale), corresponding to a recent twin-study which reported a genetic influence on the link between autistic traits and sensory sensitivity (Neufeld et al., 2021). Another possible explanation could be a common genetic liability for psychopathology influencing both autism and eating behavior (van ’t Hof et al., 2020). Our results suggest that overall eating problems in autism might not arise because of autistic core characteristics as has been hypothesized (Johnson et al., 2014), but rather that both phenomena share common genetic underpinnings. However, autistic traits were associated with scores on the subscale *Eating behavior* also within MZ-pairs, where shared environment and genetics are fully adjusted for. This could suggest a causal effect, i.e. that autistic traits contribute to the development of selective eating, or that the same non-shared environmental factors influence both.

A previous twin-study reported a shared environmental influence on the relationship between autism and behaviors related to EDs using cross-twin cross-trait associations (Råstam et al., 2013). In contrast, we did not find support for a shared environmental influence on the link between autism and total eating problems, which would have been indicated if the association found across individuals was lost within MZ- and DZ-pairs. There are several potential explanations for these differences, including that our sample was older (between 15 and 33 years compared to children aged 9 and 12 years in the study by Råstam and colleagues), and the influence of environmental and genetic factors might shift during development. Several traits show increased heritability with higher age (Bergen et al., 2007), which has been found also for eating behavior where the genetic influence on food neophobia appear to increase from early to late childhood (Cooke et al., 2007; Smith et al., 2017). We also used a broader operationalization of eating problems encompassing a multitude of issues common in autism, whereas the study by Råstam et al. (2013) focused on failure to gain weight and fear of gaining weight, which is not covered by the SWEAA. Finally, our zygosity group specific results should be interpreted with necessary caution, as confidence intervals in MZ and DZ pairs were overlapping. Future studies should investigate the link between autism and eating problems in larger twin samples, and include bivariate models yielding quantitative estimates of genetic and environmental influences common to both traits.

## Limitations

Some limitations in our study should be considered. The RATSS sample is not randomly selected from the general population, selection is based on characteristics of twin pairs, where pairs discordant or concordant for autism and ADHD are prioritized, which could affect the generalizability of the results from across-individuals analyzes. However, our results largely correspond to findings from previous studies such as elevated total eating problems among autistic individuals. While general concerns have been raised regarding the generalizability of findings in twins to singletons, studies do not find a substantially higher prevalence of autism, or higher autistic traits, among twins compared to singletons (Curran et al., 2011; Lundström et al., 2015). Furthermore, we assessed eating problems through a self-report questionnaire, requiring that participants themselves are aware of and able to report their eating behaviors. In addition, our sample included six participants with mild ID, a group for whom SWEAA has not been validated, and we cannot be certain if the questionnaire provides a valid measure of eating problems for these participants. Therefore, we excluded the participants with ID, which also excluded the outlier seen in Fig. 2b, and reran our main analyses. This did not change our main results substantially, but the across-individuals association between autistic traits and the *Simultaneous capacity* subscale was no longer significant in this subsample (see Supplementary Tables 4–7). Finally, as the SWEAA lacks items focusing on some of the main characteristics of EDs, such as fear of gaining weight, its ability to yield a valid measure of ED symptoms is limited. Also, while this measure has shown diagnostic validity in discriminating between autistic and neurotypical groups (Demartini et al., 2021; Karlsson et al., 2013; Spek et al., 2020), further validation of the SWEAA should be conducted, including assessment of concurrent validity with other well-used instruments. On the other hand, the SWEAA is one of few measures assessing a broad range of eating problems from first-hand experiences of autistic individuals, and has shown good psychometric properties (Karlsson et al., 2013).

The study was well powered to detect medium-sized associations in the across-individuals analyses but our sample was underpowered for associations corresponding to small effect sizes. Furthermore, the statistical power is reduced in the within-pair analyses and when splitting the sample in MZ- and DZ-pairs. However, the lacking associations specifically within MZ-pairs were likely not due to lacking power as our sample included more MZ-pairs than DZ-pairs (63 MZ vs. 33 DZ-pairs). While a larger sample would have increased the statistical power, the current sample of twins underwent thorough assessments, and the analyses used both parent-rated autistic traits and clinician assessment of autism diagnosis, reducing the risk of rater bias.

## Conclusions

Our results indicate that autistic traits and autism diagnosis are linked to increased eating problems among adolescents and adults, both in total eating problems and in specific problems including sensory sensitivity in mealtimes and selective eating. The link between autistic traits and total eating problems as well as specific problems with eating in social situations was especially pronounced among females, suggestive of a gender difference in the association between autism and eating behavior. Furthermore, our results point towards a possible genetic influence on the link between autism and eating problems. Our study adds to previous studies in emphasizing that eating problems are common also among autistic adolescents and adults, and provides further evidence of gender differences in eating issues associated with autism. Our findings highlight the clinical importance of assessing eating problems among autistic adolescents and adults, as well as their potentially detrimental social consequences, particularly for autistic females. Future research should investigate the impact of eating problems on quality of life, mental health and social functioning among autistic adults, as well as potential gender-related pathways suggested by our data, including whether eating problems among autistic females contribute to mental health issues or social isolation particularly.

## Supplementary Information

Below is the link to the electronic supplementary material.

Supplementary file1 (DOCX 57 KB)
