# Supplementary material for: Eating Problems in Autistic Females and Males: A Co-twin Control Study
Source: J Autism Dev Disord. 2021 Jul 22;52(7):3153–68. doi: 10.1007/s10803-021-05198-z (PMC9213283; doi:10.1007/s10803-021-05198-z)
Supplement: Supplementary file 1 — Supplementary file1 (DOCX 57 KB) [file 10803_2021_5198_MOESM1_ESM.docx]

*Interaction effect in the Mealtime surroundings subscale*

The subscale *Mealtime surroundings* was split into items focusing on eating in social contexts (six items covering difficulties in eating at school/workplace/activity center, with friends, relatives, in cafés, restaurants, and abroad) and items focusing on rituals and rigidity in mealtimes (five items), and an adjusted regression analyses was rerun for each of these two clusters separately. An interaction effect between autistic traits and gender was only found for the social items (see supplementary Table 2), where follow-up analyses showed that autistic traits and autism diagnosis predicted higher scores among females (b = 0.30, 95% CI = 0.15 to 0.45, p < 0.001; b = 17.28, 95% CI = 8.54 to 26.03, p < 0.001). For females, ADHD diagnosis also predicted increased scores on social items in both the autistic traits and autism diagnosis models, while Internalizing conditions predicted more problems on social items, and higher age and IQ less problems, in the autism diagnosis model only. For males, autistic traits were not associated with social items in the *Mealtime surroundings* subscale, while autism diagnosis predicted *less* self-reported problems on social items (b = -7.47, 95% CI = -13.41 to -1.54, p = 0.014). None of the covariates were associated with social items in this subscale for males. See Supplementary Table 1 for details.

**Supplementary Table 1.** Follow-up analyses stratified by gender for across-individuals models showing interaction effects.

|  | SWEAA total score | E. Mealtime surroundings | E. Social |
| --- | --- | --- | --- |
|  | b (95% CI) / SE / p | b (95% CI) / SE / p | b (95% CI) / SE / p |
| Females |  |  |  |
| Autistic traits | **0.14 (0.07 to 0.21) / 0.04 /**  **<0.001** | **0.20 (0.09 to 0.31) / 0.06 /**  **<0.001** | **0.30 (0.15 to 0.45) / 0.07 /**  **<0.001** |
| Age | **-0.37 (-0.63 to -0.11) / 0.13 / 0.006** | **-0.53 (-0.92 to -0.14) / 0.20 /**  **0.008** | -0.52 (-1.11 to 0.07) / 0.30 /  0.084 |
| ADHD | **8.63 (3.18 to 14.08) / 2.78 / 0.002** | **10.71 (0.95 to 20.48) / 4.98 /**  **0.032** | **16.43 (1.28 to 31.58) / 7.73 /**  **0.034** |
| Internalizing conditions | **3.59 (0.19 to 7.00) / 1.74 /**  **0.038** | 3.74 (-1.30 to 8.78) / 2.57 /  0.146 | 6.69 (-0.38 to 13.76) / 3.61 /  0.064 |
| IQ | 0.03 (-0.08 to 0.15) / 0.06 /  0.589 | -0.07 (-0.20 to 0.06) / 0.07 /  0.300 | -0.13 (-0.29 to 0.04) / 0.08 /  0.135 |
| Autism | - ^a^ | **10.98 (4.25 to 17.71) / 3.43 /**  **0.001** | **17.28 (8.54 to 26.03) / 4.46 /**  **<0.001** |
| Age | - | **-0.79 (-1.23 to -0.36) / 0.22 /**  **<0.001** | **-0.91 (-1.55 to -0.27) / 0.33 /**  **0.005** |
| ADHD | - | **14.43 (5.20 to 23.67) / 4.71 /**  **0.002** | **21.71 (7.75 to 35.67) / 7.12 /**  **0.002** |
| Internalizing conditions | - | 4.53 (-0.76 to 9.83) / 2.70 /  0.093 | **7.97 (0.49 to 15.45) / 3.82 /**  **0.037** |
| IQ | - | -0.11 (-0.24 to 0.01) / 0.07 /  0.081 | **-0.19 (-0.34 to -0.04) / 0.08 /**  **0.015** |
| Males |  |  |  |
| Autistic traits | **0.06 (0.01 to 0.11) / 0.03 /**  **0.019** | -0.01 (-0.09 to 0.07) / 0.04 /  0.871 | -0.04 (-0.12 to 0.04) / 0.04 /  0.282 |
| Age | 0.07 (-0.31 to 0.45) / 0.19 /  0.727 | -0.13 (-0.59 to 0.33) / 0.24 /  0.586 | -0.01 (-0.58 to 0.56) / 0.29 /  0.974 |
| ADHD | -0.68 (-4.36 to 3.01) / 1.88 / 0.720 | -0.04 (-5.09 to 5.01) / 2.58 /  0.987 | 1.82 (-4.91 to 8.55) / 3.44 /  0.596 |
| Internalizing conditions | 3.01 (-2.70 to 8.72) / 2.91 /  0.301 | 6.48 (-1.84 to 14.79)/ 4.24 /  0.127 | 6.69 (-1.87 to 15.25) / 4.37 /  0.126 |
| IQ | -0.06 (-0.18 to 0.06) / 0.06 / 0.342 | -0.08 (-0.20 to 0.04) / 0.06 /  0.210 | -0.13 (-0.30 to 0.03) / 0.08 /  0.109 |
| Autism | - ^a^ | -3.29 (-9.53 to 2.95) / 3.18 /  0.301 | **-7.47 (-13.41 to -1.54) / 3.03 / 0.014** |
| Age | - | -0.11 (-0.57 to 0.35) / 0.23 /  0.652 | 0.05 (-0.51 to 0.61) / 0.28 /  0.862 |
| ADHD | - | 0.12 (-4.20 to 4.43) / 2.20 /  0.958 | 1.73 (-4.15 to 7.61) / 3.00 /  0.564 |
| Internalizing conditions | - | 6.75 (-1.23 to 14.72) / 4.07 /  0.098 | 7.06 (-0.86 to 14.97) / 4.04 /  0.081 |
| IQ | - | -0.09 (-0.21 to 0.03) / 0.06 /  0.154 | -0.14 (-0.32 to 0.03) / 0.09 /  0.102 |

Follow-up analyses for females and males separately for the across-individuals models showing a significant interaction effect between autistic traits/autism and gender. b: regression coefficient; CI: confidence interval; SE: standard error. Significant results in bold (p < 0.05).

^a^As no interaction effect was found for total eating problems follow-up analyses with autism diagnosis split by gender were not conducted.

**Supplementary Table 2.** Across-individuals models for E. Social and E. non-social.

|  | E. social | | | E. non-social | | |
| --- | --- | --- | --- | --- | --- | --- |
|  | b (95% CI) | SE | p | b (95% CI) | SE | p |
| Autistic traits | **0.36 (0.22 to 0.50)** | **0.07** | **<0.001** | **0.11 (0.014 to 0.21)** | **0.05** | **0.025** |
| Gender | **7.29 (2.36 to 12.21)** | **2.51** | **0.004** | 2.98 (-1.91 to 7.86) | 2.49 | 0.232 |
| Age | -0.28 (-0.70 to 0.15) | 0.22 | 0.208 | **-0.40 (-0.71 to -0.09)** | **0.16** | **0.011** |
| ADHD | 7.07 (-0.62 to 14.75) | 3.92 | 0.071 | -0.30 (-4.72 to 4.12) | 2.25 | 0.893 |
| Internalizing conditions | **7.43 (1.95 to 12.92)** | **2.80** | **0.008** | 2.72 (-1.94 to 7.37) | 2.37 | 0.252 |
| IQ | -0.12 (-0.24 to 0.003) | 0.06 | 0.056 | 0.001 (-0.11 to 0.11) | 0.05 | 0.989 |
| Autistic traits x Gender | **-0.43 (-0.58 to -0.29)** | **0.07** | **<0.001** | -0.08 (-0.22 to 0.07) | 0.07 | 0.295 |

b: regression coefficient; CI: confidence interval; SE: standard error. Significant results in bold

(p < 0.05).

**Supplementary Table 3.** Within-pair associations between autistic traits and separate SWEAA subscales.

| Model | MZ | DZ |  |  |  |
| --- | --- | --- | --- | --- | --- |
|  | Autistic traits | Autistic traits | ADHD | Internalizing conditions | IQ |
|  | b / SE / p | b / SE / p | b / SE / p | b / SE / p | b / SE / p |
| 1. Perception | 0.04 / 0.10 /  0.708 | **0.12 / 0.06 / 0.038** | 5.61 / 4.94/  0.256 | -1.77 / 3.66 /  0.629 | 0.11 / 0.12 /  0.333 |
| 1. Eating behavior | **0.23 / 0.09/**  **0.013** | **0.22 / 0.07 / 0.001** | 1.60 / 2.79 /  0.568 | -2.12 / 2.45 /  0.386 | 0.16 / 0.11 /  0.147 |
| 1. Mealtime surroundings | 0.05 / 0.09 /  0.563 | **0.13 / 0.07 / 0.049** | 0.46 / 3.76 /  0.903 | 1.40 / 2.25 /  0.534 | -0.08 / 0.09 /  0.402 |
| 1. Social situation at mealtime | 0.07 / 0.08 /  0.431 | **0.23 / 0.08 / 0.005** | 1.25 / 5.00 /  0.802 | 2.45 / 2.59 /  0.343 | -0.02 / 0.11 /  0.836 |
| 1. Simultaneous capacity | -0.04 / 0.06 /  0.544 | 0.11 / 0.07 / 0.086 | 12.07 / 7.18 / 0.093 | 2.88 / 2.74 /  0.294 | 0.11 / 0.11 /  0.291 |

b: regression coefficient; SE: standard error. Significant results in bold (p < 0.05).

**Supplementary Table 4.** Across-individuals associations between autism and total SWEAA score excluding participants with ID (n = 186).

|  | Unadjusted model | | | Adjusted model | | |
| --- | --- | --- | --- | --- | --- | --- |
|  | b (95% CI) | SE | p | b (95% CI) | SE | p |
| Autistic traits | **0.14 (0.10 to 0.19)** | **0.02** | **<0.001** | **0.17 (0.10 to 0.24)** | **0.04** | **<0.001** |
| Gender |  |  |  | 3.14 (-0.01 to 6.30) | 1.61 | 0.051 |
| Age |  |  |  | -0.17 (-0.39 to 0.05) | 0.11 | 0.123 |
| ADHD |  |  |  | 2.02 (-1.68 to 5.72) | 1.89 | 0.284 |
| Internalizing conditions |  |  |  | **4.38 (1.18 to 7.57)** | **1.63** | **0.007** |
| IQ |  |  |  | 0.01 (-0.08 to 0.10) | 0.05 | 0.789 |
| Autistic traits x Gender |  |  |  | **-0.11 (-0.18 to -0.03)** | **0.04** | **0.007** |
| Autism diagnosis | **7.41 (3.53 to 11.29)** | **1.98** | **<0.001** | **9.40 (3.99 to 14.82)** | **2.76** | **<0.001** |
| Gender |  |  |  | 0.68 (-2.13 to 3.50) | 1.44 | 0.635 |
| Age |  |  |  | **-0.33 (-0.57 to -0.09)** | **0.12** | **0.007** |
| ADHD |  |  |  | 3.41 (-0.46 to 7.28) | 1.97 | 0.084 |
| Internalizing conditions |  |  |  | **5.01 (1.69 to 8.33)** | **1.69** | **0.003** |
| IQ |  |  |  | -0.01 (-0.10 to 0.08) | 0.04 | 0.822 |
| Autism x Gender |  |  |  | -6.00 (-13.99 to 1.99) | 4.08 | 0.141 |

b: regression coefficient; CI: confidence interval; SE: standard error. Significant results in bold

(p < 0.05).

**Supplementary Table 5.** Across-individuals associations between autistic traits and SWEAA scores excluding six participants with ID (n = 186).

|  | Autistic traits | Gender | Age | ADHD | Internalizing conditions | IQ | Autistic traits x Gender |
| --- | --- | --- | --- | --- | --- | --- | --- |
|  | b / SE / p | b / SE / p | b / SE / p | b / SE / p | b / SE / p | b / SE / p | b / SE / p |
| 1. Perception | **0.23 /**  **0.07 /**  **0.001** | 0.09 /  2.41 /  0.972 | -0.32 / 0.19 / 0.095 | 4.15 /  3.23 /  0.200 | 2.39 / 2.16 / 0.269 | 0.10 /  0.08 /  0.219 | -0.14 / 0.08 /  0.082 |
| 1. Motor control | 0.08 / 0.04 / 0.026 | 3.60 /  2.28 /  0.115 | -0.16 / 0.14 / 0.277 | -0.18/  2.68 /  0.948 | 6.16 / 2.04 / 0.003 | 0.02 /  0.04 /  0.708 | -0.05 / 0.06 /  0.445 |
| 1. Purchase of food | 0.08 /  0.07 /  0.253 | 2.96 /  3.84 /  0.441 | 0.89 / 0.35 / 0.010 | 4.12 /  3.74 /  0.270 | 2.70 / 3.59 / 0.453 | 0.09 /  0.10 /  0.372 | -0.15 / 0.08 /  0.065 |
| 1. Eating behavior | **0.24 /**  **0.07 / <0.001** | 7.21 /  3.27 /  0.027 | -0.06 / 0.22 / 0.792 | 1.47 /  3.45 /  0.671 | 4.79 / 3.10 / 0.122 | 0.03 /  0.08 /  0.679 | -0.15 / 0.10 /  0.133 |
| 1. Mealtime surroundings | **0.21 /**  **0.05 / <0.001** | 4.41 /  2.05 /  0.031 | -0.34 / 0.16 / 0.030 | 2.37 /  2.57 /  0.358 | 6.26 / 2.31 / 0.007 | -0.04 / 0.05 /  0.450 | **-0.22 / 0.06 /**  **<0.001** |
| 1. Social situation at mealtime | **0.25 /**  **0.05 / <0.001** | **8.01 /**  **2.65 /**  **0.002** | -0.24 / 0.16 / 0.133 | -2.40 / 2.73 /  0.380 | 4.90 / 2.04 / 0.016 | -0.06 / 0.06 /  0.335 | -0.01 / 0.07 /  0.865 |
| 1. Other behavior associated with disturbed eating | 0.01 /  0.03 /  0.782 | -2.29 / 1.78 / 0.198 | -0.08 / 0.11 / 0.474 | 2.23 /  2.05 /  0.278 | 1.95 / 1.56 / 0.212 | -0.04 / 0.03 /  0.259 | -0.01 / 0.03 / 0.684 |
| 1. Hunger / Satiety | 0.25 /  0.09 /  0.005 | -0.09 / 4.36 /  0.984 | -0.53 / 0.33 / 0.104 | 14.50 / 7.07 / 0.04 | 9.83 / 4.08 / 0.016 | 0.20 /  0.11 /  0.059 | -0.23 / 0.11 /  0.039 |
| 1. Simultaneous capacity | 0.12 /  0.04 /  0.007 | 1.73 /  1.58 /  0.274 | -0.19 / 0.14 / 0.181 | 8.11 /  4.67 /  0.082 | 3.13 / 2.37 / 0.186 | 0.02 / 0.07 / 0.765 | -0.12 / 0.07 /  0.080 |
| 1. Pica | 0.01 /  0.01 /  0.699 | -1.04 / 1.81 /  0.564 | -0.01 / 0.06 / 0.809 | -1.67 / 1.97 /  0.397 | 0.11 / 0.89 / 0.901 | -0.03 / 0.02 /  0.073 | 0.06 / 0.06 /  0.314 |

b: regression coefficient; SE: standard error. Significant results in bold (p < 0.005).

**Supplementary Table 6.** Associations between autism diagnosis and SWEAA total score and subscales excluding participants with ID (n = 186).

|  | Autism | Gender | Age | ADHD | Internalizing conditions | IQ | Autism x Gender |
| --- | --- | --- | --- | --- | --- | --- | --- |
|  | b / SE / p | b / SE / p | b / SE / p | b / SE / p | b / SE / p | b / SE / p | b / SE / p |
| A. Perception | **14.68 /**  **5.72 /**  **0.010** | -3.60 / 2.02 / 0.075 | **-0.54 / 0.20 / 0.006** | 5.64 / 3.31 / 0.089 | 3.17 / 2.15 / 0.141 | 0.08 / 0.08  / 0.297 | -5.82 / 7.53 / 0.439 |
| D. Eating behavior | **13.84 /**  **5.35 /**  **0.010** | 3.38 / 2.70 / 0.211 | -0.29 / 0.23 / 0.206 | 3.34 / 3.52 / 0.342 | 5.62 / 3.10 / 0.070 | 0.01 / 0.08  / 0.908 | -6.07 / 8.38 / 0.469 |
| E. Mealtime surroundings | **10.72 /**  **3.48 /**  **0.002** | -0.20 / 1.87 / 0.914 | **-0.50 / 0.17 / 0.004** | 3.49 / 2.50 / 0.163 | **7.22 / 2.40 / 0.003** | -0.07 / 0.05 / 0.157 | **-14.48 /**  **4.96 / 0.004** |
| F. Social situation at mealtime | **14.73 /**  **4.09 /**  **<0.001** | **7.94 / 1.95 / <0.001** | **-0.51 / 0.15 / 0.001** | 0.94 / 2.81 / 0.737 | **5.77 / 2.12 / 0.007** | -0.10 / 0.06 / 0.093 | -3.82 / 5.81 / 0.511 |

b: regression coefficient; SE: standard error. Significant results in bold (p < 0.05).

**Supplementary Table 7.** Within-pair association between autistic traits and SWEAA total score excluding participants with ID (n = 182).

|  | MZ | DZ |  |  |  |
| --- | --- | --- | --- | --- | --- |
|  | Autistic traits | Autistic traits | ADHD | Internalizing conditions | IQ |
|  | b (95%) / SE / p | b (95%) / SE / p | b (95%) / SE / p | b (95%) / SE / p | b (95%) / SE / p |
| SWEAA total score | 0.03 (-0.09 to 0.15) / 0.06 / 0.646 | **0.13 (0.05 to 0.20) /**  **0.04 / <0.001** | 2.93 (-2.39 to 8.25) / 2.71 / 0.280 | 1.95 (-1.34 to 5.24) / 1.68 / 0.245 | 0.04 (-0.08 to 0.16) / 0.06 / 0.526 |

Twin pairs that were incomplete after excluding participants with ID are excluded in this model, as it requires both twins in a pair. b: regression coefficient; CI: confidence interval; SE: standard error. Significant results in bold (p < 0.05).
